# The EKG in cardiac MRI: what the technologist needs to know

**DOI:** 10.1186/1532-429X-16-S1-T4

**Published:** 2014-01-16

**Authors:** Emer Sonnex, Richard Coulden

**Affiliations:** 1 Department of Radiology & Diagnostic Imaging, University of Alberta Hospital, Edmonton, Alberta, Canada

## Background

In routine cardiac MRI (CMRI) practice, radiological technologists are expected to optimize and utilize EKGs in gating and physiological monitoring. Many cardiac sequences are critically dependent on heart rate and rhythm and EKG gating problems lead to poor or non-diagnostic images. The technologist's syllabus does not routinely include formal EKG recognition or CMRI trouble-shooting techniques.

## Methods

In this educational exhibit, we describe common EKG rhythms and discuss imaging parameter options for overcoming magneto-hemodynamic effects, dealing with high or low heart rates and abnormal rhythms.

## Results

An image sequence/EKG rhythm quick algorithm is included for reference and will be the basis of the exhibit.

## Conclusions

Basic EKG rhythm recognition and imaging optimization tips are the building blocks of good cardiac MRI - something every good CMRI technologist should know but are rarely formally taught.

## Funding

None.

**Figure 1:**
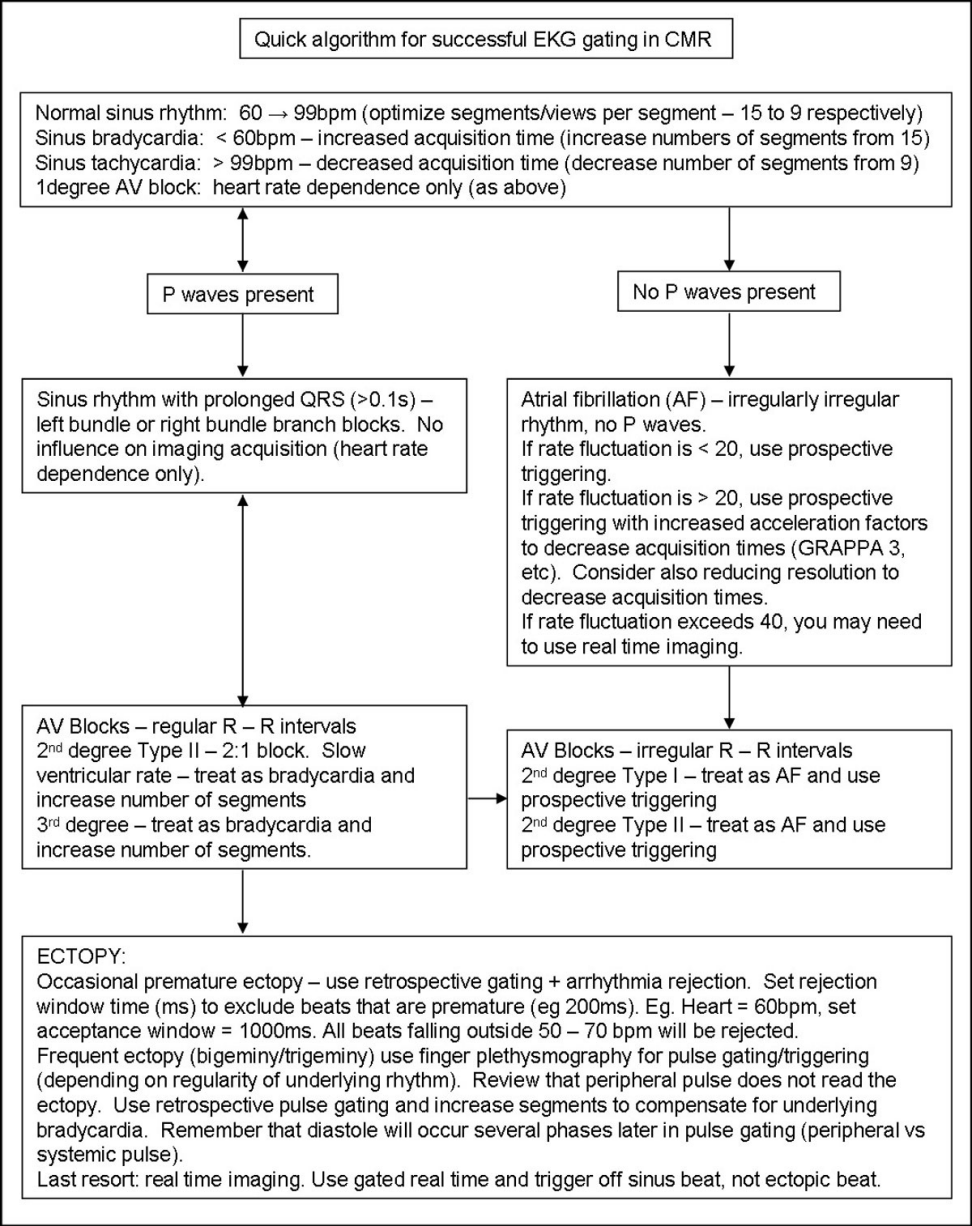
**Quick algorithm for successful EKG gating in CMRI**.

